# Supplementary material for: Inhibitors of trehalose-6-phosphate synthase activity in fungal pathogens compromise thermal tolerance pathways
Source: mBio. 2025 Aug 25;16(10):e01795-25. doi: 10.1128/mbio.01795-25 (PMC12505902; doi:10.1128/mbio.01795-25)
Supplement: Supplemental Figures — Figures S1 to S12. [file mbio.01795-25-s0001.pdf]

A

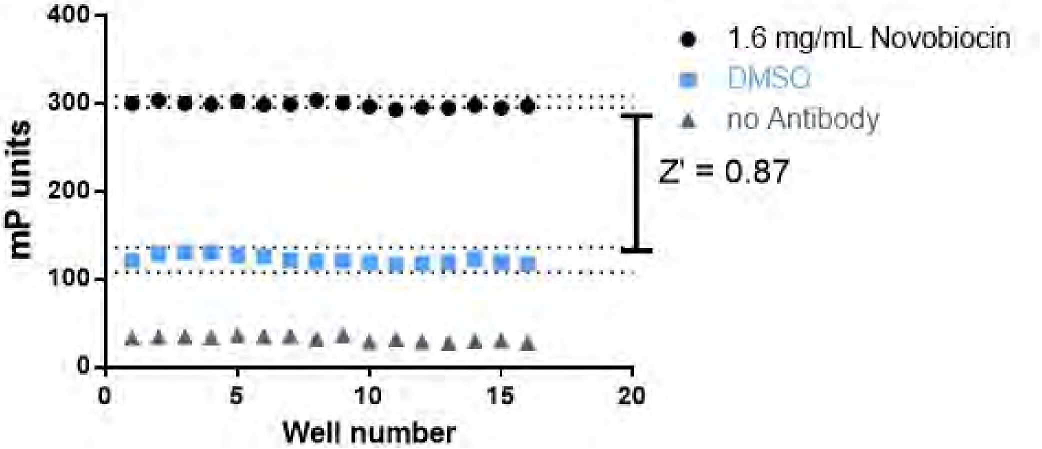

400
300
200
100
0
mP units
0
5
10
15
20
Well number
1.6 mg/mL Novobiocin
DMSO
no Antibody
Z' = 0.87

B

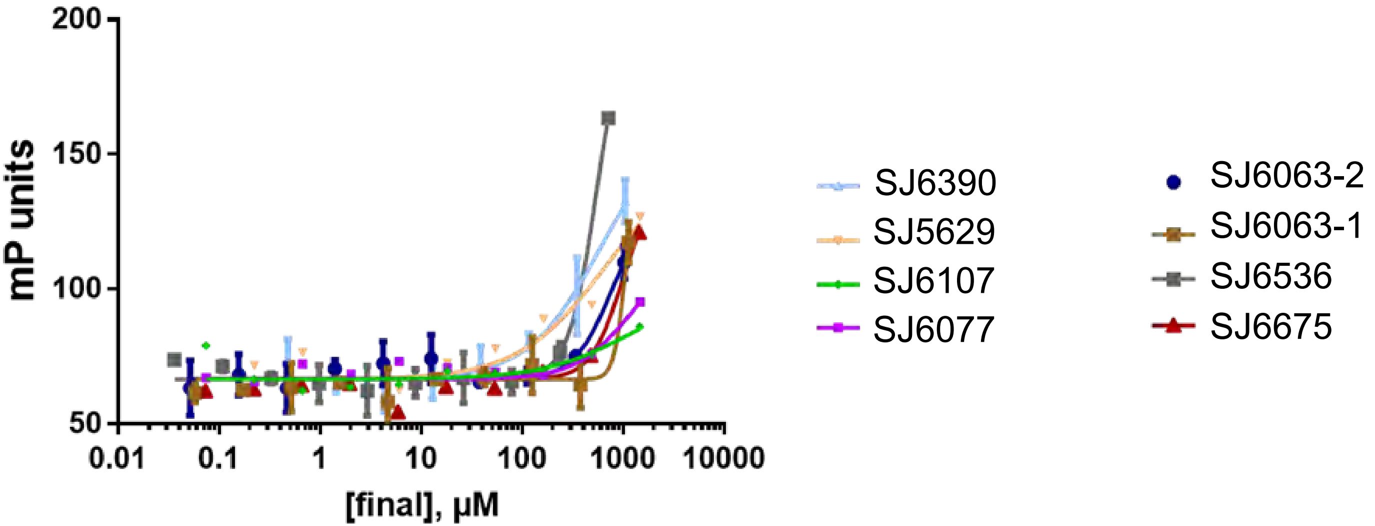

200
150
100
50
mP units
0.01
0.1
1
10
100
1000
10000
[final], µM
SJ6390
SJ5629
SJ6107
SJ6077
SJ6063-2
SJ6063-1
SJ6536
SJ6675

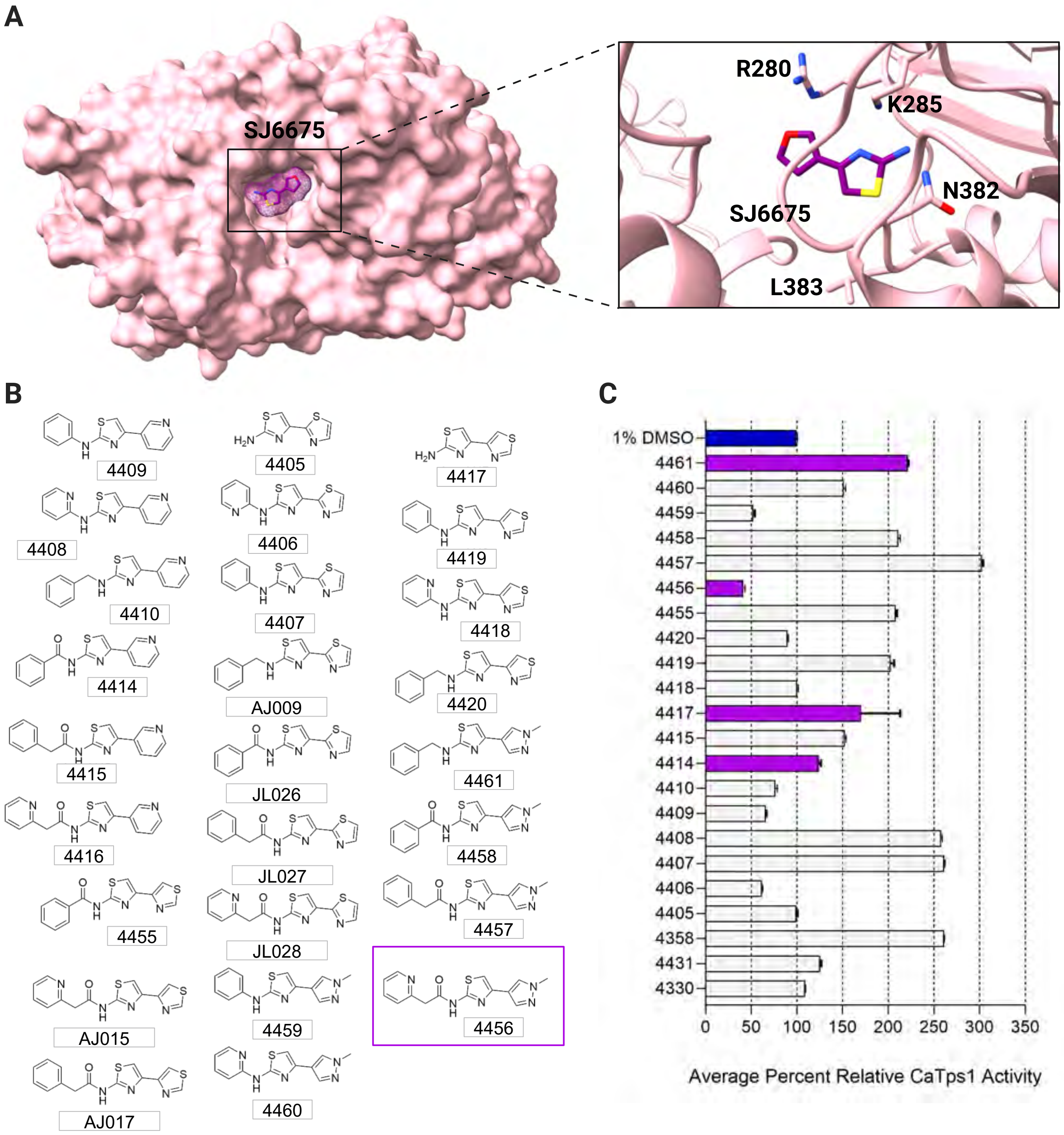
A
SJ6675
R280
K285
N382
SJ6675
L383
B
4409
4408
4410
4414
4415
4416
4455
AJ015
AJ017
4405
4406
4407
AJ009
JL026
JL027
JL028
4459
4460
4417
4419
4418
4420
4461
4458
4457
4456
C
1% DMSO
4461
4460
4459
4458
4457
4456
4455
4420
4419
4418
4417
4415
4414
4410
4409
4408
4407
4406
4405
4358
4431
4330
0 50 100 150 200 250 300 350
Average Percent Relative CaTps1 Activity

A

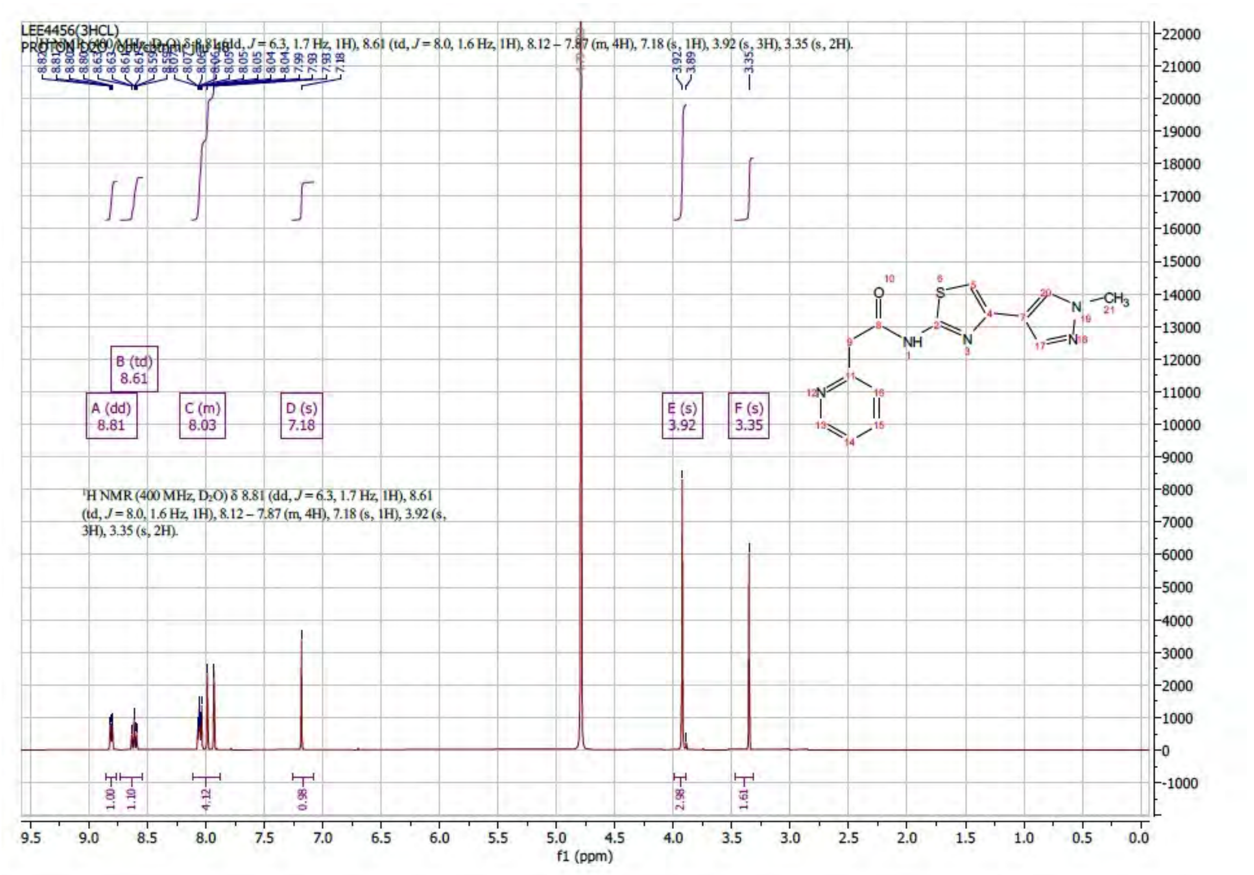

B

Element	Theory	Found	
C	41.14	42.56	42.48
H	3.95	4.51	4.54
N	17.13	17.32	17.34
Cl	26.02	19.46	26.02

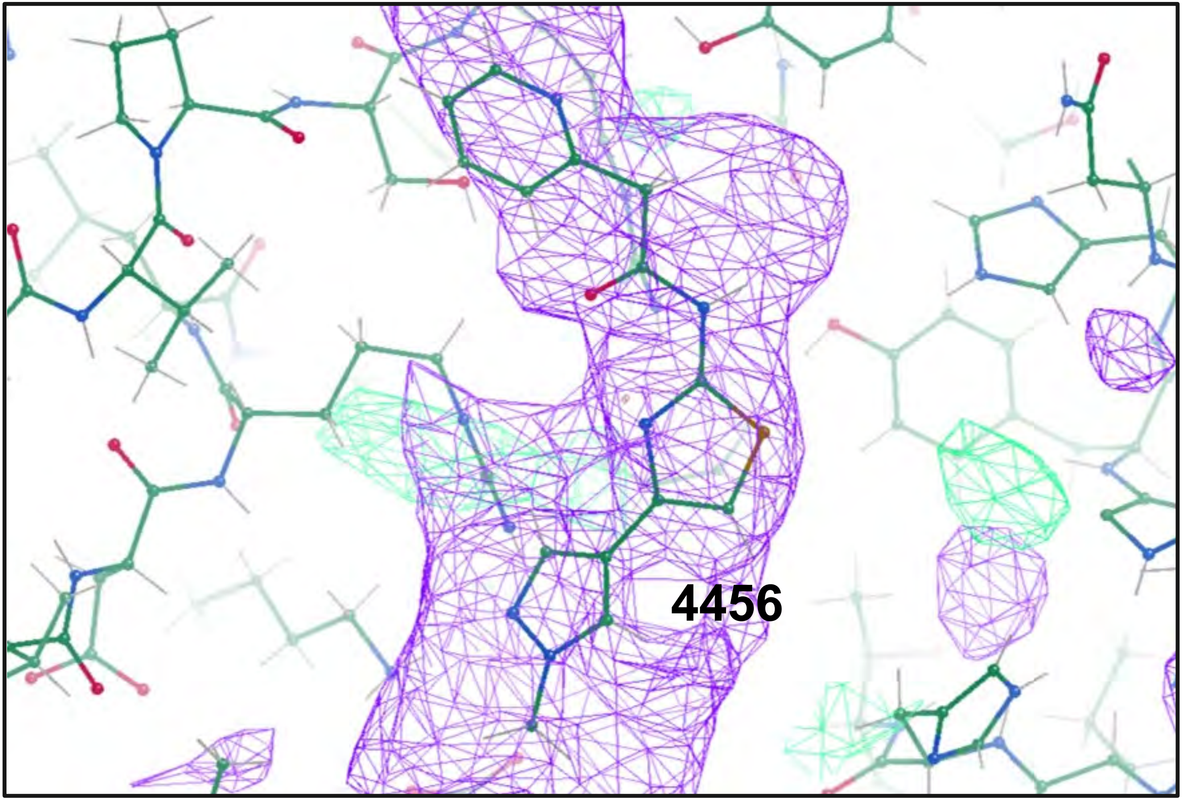
4456

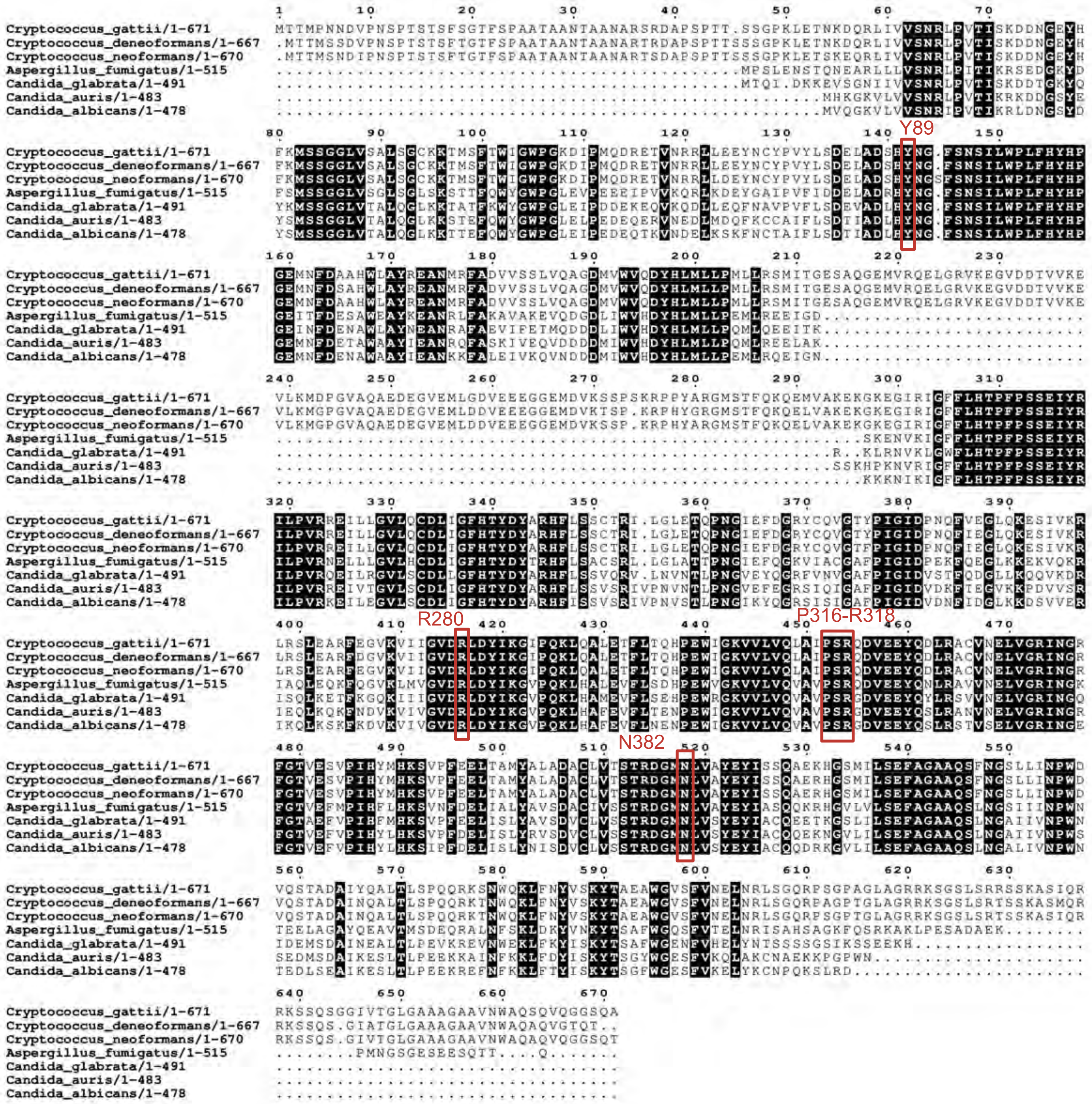
Cryptococcus_gattii/1-671
Cryptococcus_deneoformans/1-667
Cryptococcus_neoformans/1-670
Aspergillus_fumigatus/1-515
Candida_glabrata/1-491
Candida_auris/1-483
Candida_albicans/1-478
Y89
R280
P316-R318
N382

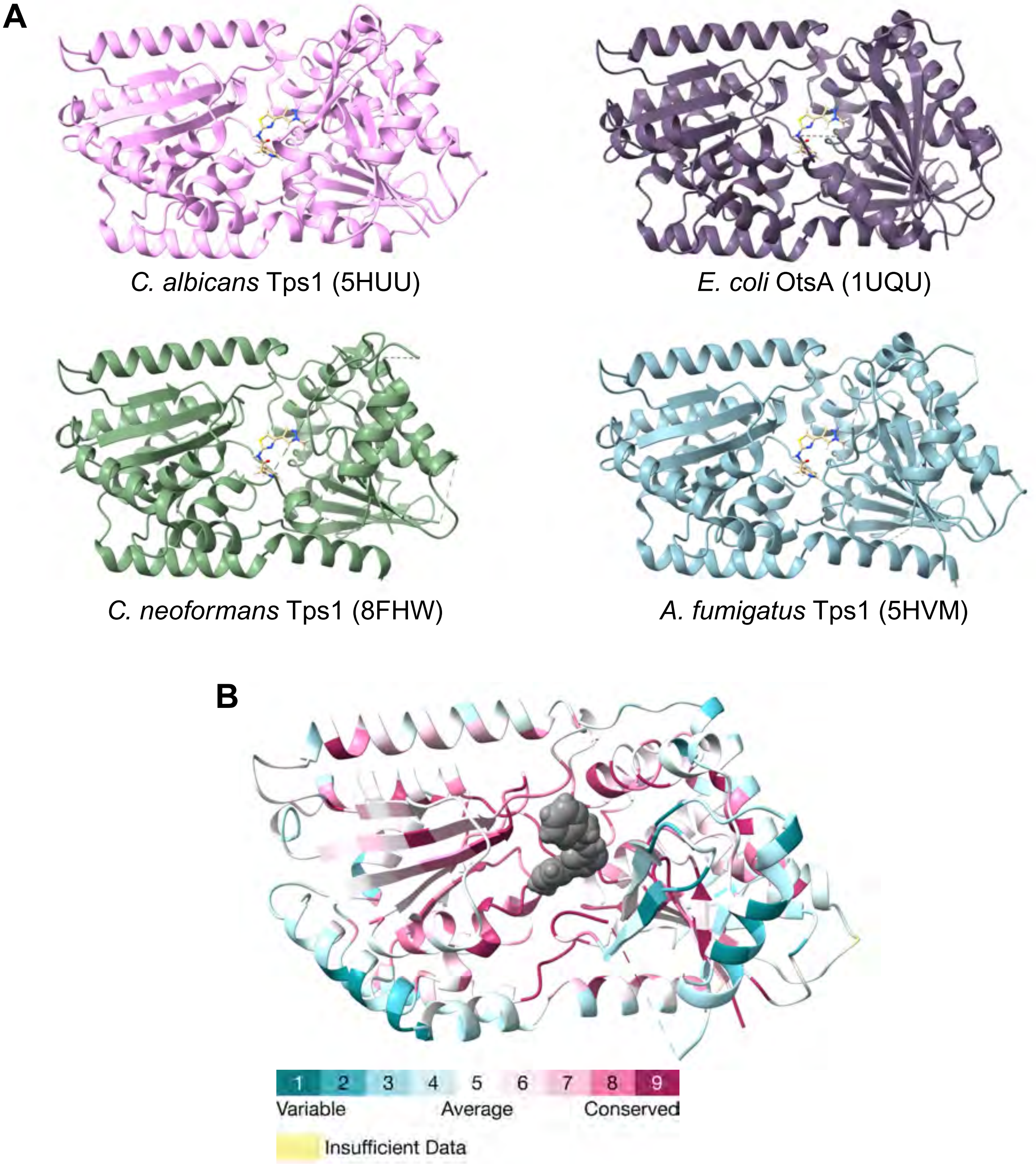
A
C. albicans Tps1 (5HUU)
E. coli OtsA (1UQU)
C. neoformans Tps1 (8FHW)
A. fumigatus Tps1 (5HVM)
B
1
2
3
4
5
6
7
8
9
Variable
Average
Conserved
Insufficient Data

A

Fungal Species	MIC_{80} (mM)
C. albicans	ND
C. auris	> 13
C. glabrata	ND
C. neoformans	ND
C. deneoformans	> 13
C. gattii	ND

B

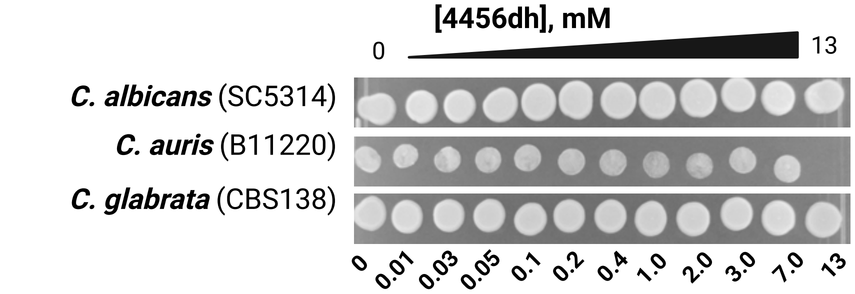

C

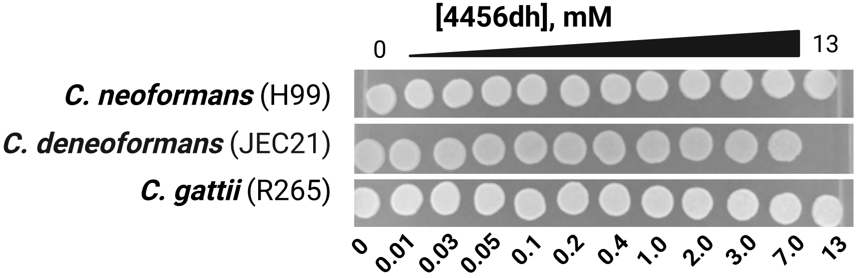

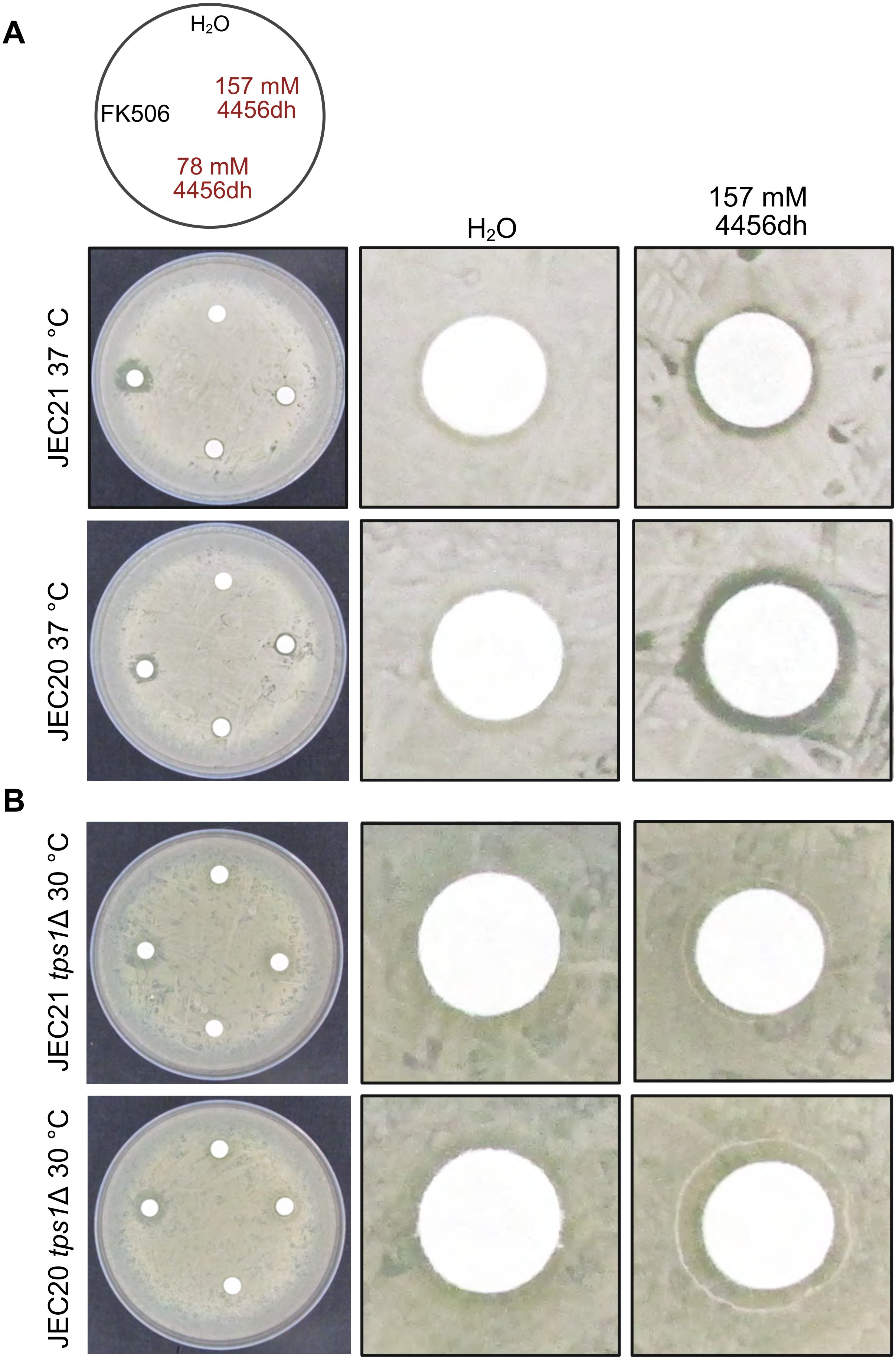

A
H_2O
157 mM 4456dh
FK506
78 mM 4456dh
H_2O
157 mM 4456dh
JEC21 37 °C
JEC20 37 °C
B
JEC21 *tps1*Δ 30 °C
JEC20 *tps1*Δ 30 °C

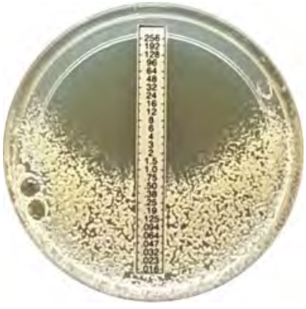

No 4456dh

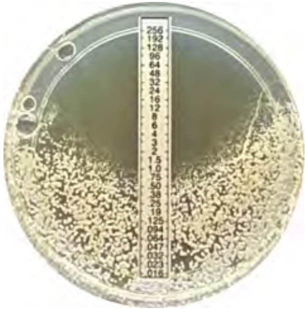

0.08 mM 4456dh

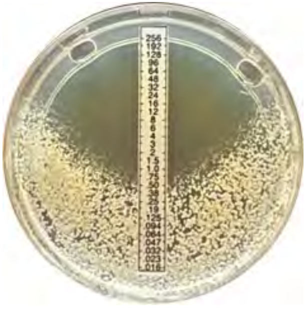

0.16 mM 4456dh

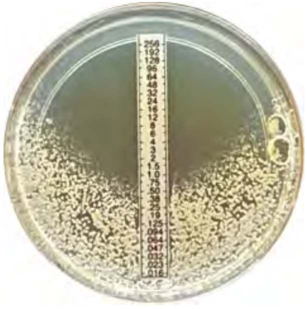

0.65 mM 4456dh

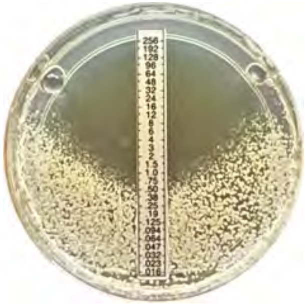

0.31 mM 4456dh

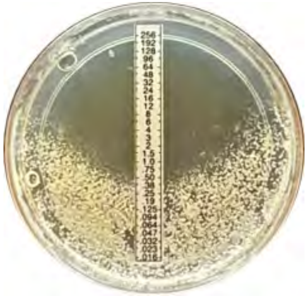

1.25 mM 4456dh

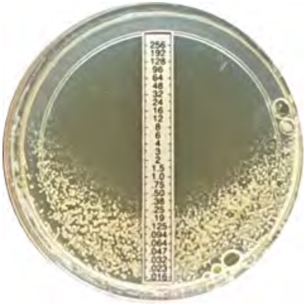

2.5 mM 4456dh

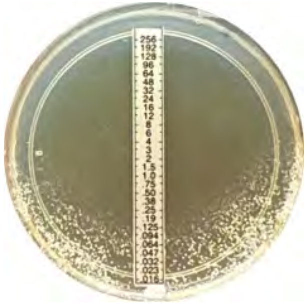

5 mM 4456dh

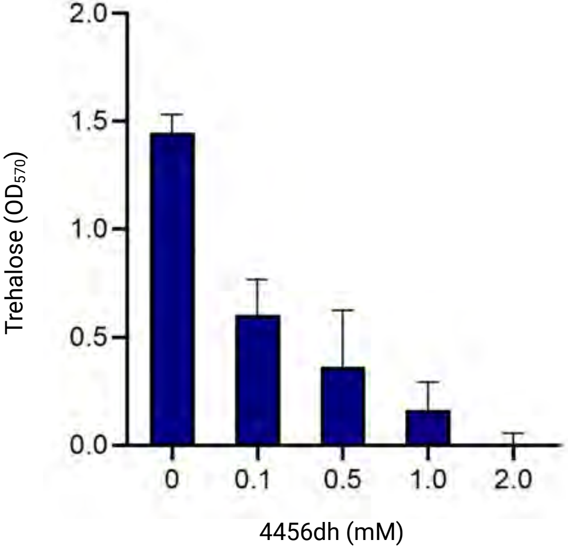
Trehalose (OD_{570})
2.0
1.5
1.0
0.5
0.0
0
0.1
0.5
1.0
2.0
4456dh (mM)

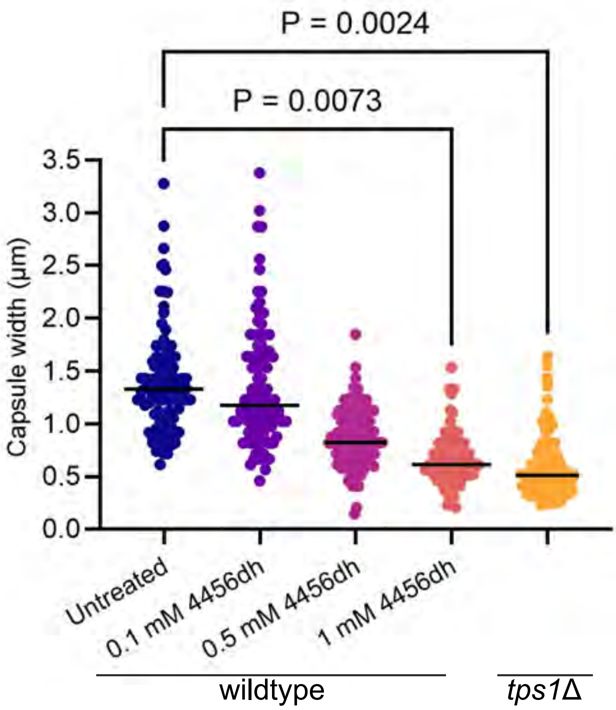

P = 0.0024
P = 0.0073
3.5
3.0
2.5
2.0
1.5
1.0
0.5
0.0
Capsule width (µm)
Untreated
0.1 mM 4456dh
0.5 mM 4456dh
1 mM 4456dh
wildtype
tps1Δ

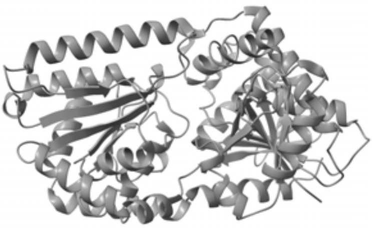

C. albicans Tps1 (5HUU)

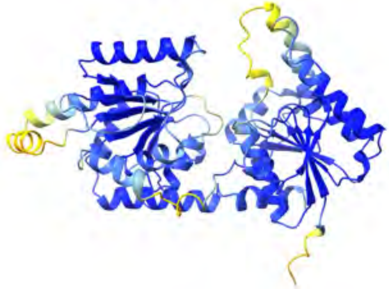

Q59LF2 Alpha-1,3/1,6-mannosyltransferase Alg2

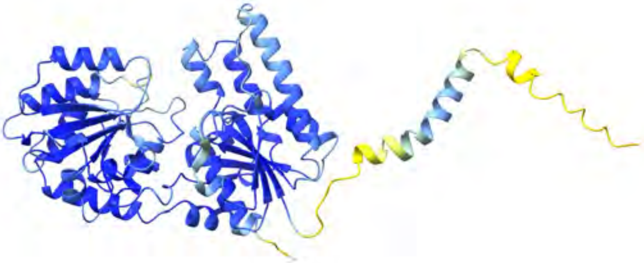

Q59Q79 Chitobiosyldiphosphodolichol beta mannosyltransferase

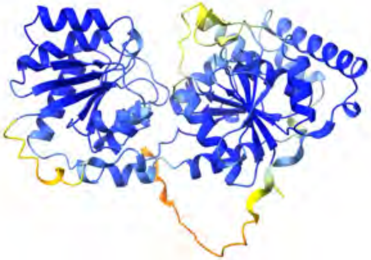

Q5A6R7 Glcnac-Pi Synthesis protein
