# Supplementary material for: Inhibitors of trehalose-6-phosphate synthase activity in fungal pathogens compromise thermal tolerance pathways
Source: mBio. 2025 Aug 25;16(10):e01795-25. doi: 10.1128/mbio.01795-25 (PMC12505902; doi:10.1128/mbio.01795-25)
Supplement: Supplemental material — Supplemental text, figure legends, and tables. [file mbio.01795-25-s0002.pdf]

Supplemental Material

Inhibitors of trehalose-6-phosphate synthase activity in fungal pathogens compromise thermal tolerance pathways

Yi Miao[a*1], Vikas Yadav[b1], William Shadrick[c^], Jiuyu Liu[c], Alexander R. Jenner[c], Connie B. Nichols[bd], Clifford Gee[c@], Martin Schäfer[a%], Jennifer L. Tenor[d], John R. Perfect[b,d], Richard E. Lee[c], Richard G. Brennan[a#], and Erica J. Washington[a,b#]

[a]Department of Biochemistry, Duke University School of Medicine, Durham, North Carolina, USA

[b]Department of Molecular Genetics and Microbiology, Duke University School of Medicine, Durham, North Carolina, USA

[c]Department of Chemical Biology and Therapeutics, St. Jude Children's Research Hospital, Memphis, Tennessee, USA

[d]Division of Infectious Diseases, Department of Medicine, Duke University School of Medicine, Durham, North Carolina, USA

#Address correspondence to: erica.washington1@duke.edu and richard.brennan@duke.edu

*Present address: Division of Life Science, The Hong Kong University of Science and Technology, Clear Water Bay, Kowloon, Hong Kong SAR, China

^Present address: Incyte Corporation, Wilmington, Delaware

@Present address: Department of Chemistry and Biochemistry, Creighton University, Omaha, Nebraska, USA

%Present address: GCP-Service International Ltd & Co. KG, Bremen, Germany

[1] co-first authors

Supplemental Methods and Materials

Synthesis of N-(4-(1-methyl-1H-pyrazol-4-yl)thiazol-2-yl)-2-(pyridin-2-yl)acetamide Dichloride Hydrate(4456).

To a stirred solution of 4-(1-methyl-1H-pyrazol-4-yl)thiazol-2-amine (**1,** 1.75 g, 9.71 mmol) in dry DMF (20mL) at room temperature, a single portion of 2-(pyridin-2-yl)acetic acid hydrochloride (**2**, 3.71 g, 21.36 mmol) and HBTU (8.10 g, 21.36 mmol) was added. The resulting mixture was stirred at room temperature for 5 minutes, followed by the addition of DIPEA (5.59 g, 48.50 mmol). The reaction was stirred for 1 hour before being partitioned between dichloromethane and water. The organic layer was washed with brine, dried over Na_2SO_4 and the solvent was removed under reduced pressure. The residue was redissolved in ethyl acetate (EA), and a pale solid was obtained by filtration (1.25g, 43.0%). To convert the product to its dichloride form, 20 mL of 1M HCl in MeOH was added to the suspended solution of **4456** (1.25g) in MeOH. Once the suspending solution became clear, the solvent was evaporated, and the residue was treated with EA. The solid was collected by filtration as **4456** dichloride hydrate (1.66g, 100%). ^{1}H NMR (400 MHz, D_2O) δ 8.81 (dd, J = 6.3, 1.7 Hz, 1H), 8.61 (td, J = 8.0, 1.6 Hz, 1H), 8.12 – 7.87 (m, 4H), 7.18 (s, 1H), 3.92 (s, 3H), 3.35 (s, 2H). Anal. Calcd for $C_{14}H_{13}N_5OS$ H_2O 2.2HCl: C 42.29, H 4.36, N 17.62, Cl 19.62; Found C 42.52, H 4.53, N 17.33, Cl 19.46.

Chemistry

4-(1-methyl-1H-pyrazol-4-yl)thiazol-2-amine (**1**) was purchased from Enamine. ^{1}H NMR spectra were recorded on a Bruker 400 MHz NMR spectrometer. Chemical shifts (δ) are reported in parts per million relative to the residual solvent peak or internal standard (tetramethylsilane) and coupling constants (J) are reported in hertz (Hz). The purity of the products was confirmed by UPLC/MS (the Waters Acquity). Elemental analysis was tested by Atlantic Microlab Inc.

Expression and Purification of *C. albicans* Tps1

C. albicans Tps1 (CaTps1) was purified as previously described (1). Briefly, the full-length *TPS1* gene from *C. albicans* strain SC5314 was codon-optimized for expression in *E. coli* (Genscript) and cloned into the pET-28a vector, which contains an N-terminal 6xHis affinity tag followed by a thrombin cleavage site. The construct was transformed into BL21(DE3)pLysS cells (Life Technologies, Inc.) and induced with 0.5 mM isopropyl β-D-1-thiogalactopyranoside (IPTG) at 15 °C overnight. Supernatants from lysed cultures were loaded onto a nickel column (Ni-NTA, Qiagen) and washed in a buffer containing 50 mM Tris-HCl pH 8.0, 300 mM NaCl, 5 mM $MgCl_2$, 5% glycerol and 5 mM imidazole. The protein was eluted with increasing amounts of imidazole in the wash buffer. The fractions containing 6xHis-CaTps1 were pooled, reduced to 5 mL and purified further using S200 size exclusion column chromatography (HiLoad 26/600 Superdex 200pg, Cytiva) in a precooled buffer containing 20 mM Tris-HCl pH 8.0, 200 mM NaCl, 5% glycerol and 1 mM β-mercaptoethanol. 5 mL fractions from the size exclusion column containing 6xHis-CaTps1, as determined by SDS-PAGE analysis, were pooled and concentrated using 30K MWCO Amicon Ultra concentrators (Millipore) to 1 mg/mL for downstream applications.

Expression and Purification of *C. neoformans* Tps1

C. neoformans Tps1 (CnTps1) was purified as previously described (2). Briefly, the full-length *TPS1* gene from *C. neoformans* strain H99 was codon-optimized for expression in *E. coli* (Genscript) and subcloned using ligation-independent cloning into pMCSG7 (3). The construct was transformed into *E. coli* OverExpress C41(DE3) chemically competent cells engineered for high protein expression (Sigma). Supernatants from lysed cultures, induced with 0.5 mM isopropyl β-D-1-thiogalactopyranoside (IPTG), were loaded onto a nickel column (Ni-NTA, Qiagen) and washed in a buffer containing 50 mM Tris-HCl pH 8.0, 300 mM NaCl, 5 mM $MgCl_2$, 5% glycerol

and 5 mM imidazole. The protein was eluted with increasing amounts of imidazole in the wash buffer. The fractions containing 6xHis-CnTps1 were pooled, reduced to 5 mL and purified further using S200 size exclusion column chromatography (HiLoad 26/600 Superdex 200pg, Cytiva) using a precooled buffer containing 20 mM Tris-HCl pH 8.0, 300 mM NaCl, 5% glycerol and 2 mM β-mercaptoethanol. 5 mL fractions from the size exclusion column containing 6xHis-CnTps1, as determined by SDS-PAGE analysis, were pooled and concentrated to 1 mg/mL for downstream applications.

Microscale Thermophoresis Assays

Recombinant and 6xHis-tagged CaTps1 and CnTps1 were purified according to the procedures described above. CaTps1 and CnTps1 were diluted to 5 µM in buffer containing (SEC buffer with 0.05% Tween20). For the dilution series, a 100 µM solution of 4456dh was prepared using the same buffer. The stock solution was used for the 16-step serial dilution in the buffer, with a final volume of 10 µL of 4456dh in each reaction mixture of the dilution series. 10 µL of protein was added to the 16 vials and samples were mixed by pipetting up and down. Reactions were incubated overnight at room temperature. Samples were loaded into Monolith NT LabelFree Premium capillaries. MST was performed at Medium MST power for both CaTps1 and CnTps1. The data were acquired with MO.Control 1.5.3 (NanoTemper Technologies GmbH). Recorded data were analyzed with MO.Affinity Analysis 2.2.7 (NanoTemper Technologies GmbH). The MST on-time that yielded that highest signal-to-noise ratio was used for the K_d determination, where K_d is the equilibrium dissociation constant.

Thermal Shift Assays

6xHis-CaTps1 and 6xHis-CnTps1 (2 µM) were individually incubated with 4456dh (100 µM) in 20 mM Tris-HCl pH 8.0, 300 mM NaCl, 5% glycerol and 2 mM β-mercaptoethanol buffer containing 2X Glo-melt dye. Thermal denaturation was measured using an RT-PCR thermocycler (BioRad) between 25 °C and 95 °C at 0.5 °C increments. The melting temperatures of each sample were determined by identifying the inflection point of the derivative data.

Tps1 Enzyme Activity Assay

The catalytic activity of *C. albicans* Tps1 and *C. neoformans* Tps1 was measured via a continuous enzyme-coupled assay as previously reported (4). Briefly, either 6xHis-CaTps1 or 6xHis-CnTps1 were concentrated in a buffer containing 20 mM Tris-HCl pH 8.0, 300 mM NaCl, 5% glycerol and 2 mM β-mercaptoethanol. The assay was performed in an assay buffer containing 50 mM HEPES pH 7.8, 100 mM KCl, 5 mM $MgCl_2$ and 2 mM DTT. Final concentrations of 3 µM protein were combined with 1 mM UDPG and 1 mM G6P. Activity assays were performed in clear, flat-bottomed 96-well microtiter plates and the decrease in absorbance at 340 nm during the initial reaction rate was recorded using a plate reader (Tecan). The decrease in absorbance was analyzed for the first 200s of the kinetic reaction.

Minimum Inhibitory Concentration Assay

Wild-type *C. albicans* strains SC5314, wild-type *C. glabrata* CBS138 and wild-type *C. auris* B11220 were cultured overnight in YPD (yeast-peptone-dextrose) medium at 30 °C. Growth of the overnight cultures was quantified by measuring the OD_{600} and corrected for the background absorbance of the medium. Antifungal potency for 4456dh was assessed by minimum inhibitory concentration (MIC) dose-response assays performed under standard Clinical and Lab Standards Institute (CLSI) conditions. A volume of 125 µL from each diluted cell suspension was dispensed into the wells of 96-well flat-bottom microtiter plates (Corning). An additional 25 µL of 4456dh from a series of dilutions from the 157 mM stock solution prepared in water was added to each well. Following preparation, the plates were incubated at the either 30 °C or 42 °C, as indicated, for 48

hours. All dose-response assays were performed in biological triplicate with a minimum of three technical replicates. Growth, as determined by a reading of absorbance at 600 nm, was normalized to untreated controls and was corrected for the absorbance of 4456dh in the YPD medium. Growth was plotted as a heat map using Excel.

Wild-type *C. neoformans* strains H99, wild-type *C. deneoformans* JEC21 and wild-type *C. gattii* R265 were cultured overnight in YPD (yeast-peptone-dextrose) medium at 30 °C. Growth of the overnight cultures was quantified by measuring the OD_{600} and corrected for the background absorbance of the medium. Antifungal potency for 4456dh was assessed by minimum inhibitory concentration (MIC) dose-response assays performed under standard Clinical and Lab Standards Institute (CLSI) conditions. A volume of 125 µL from each cell suspension was dispensed into the wells of 96-well flat-bottom microtiter plates (Corning). An additional 25 µL of 4456dh from a series of dilutions from the 157 mM stock solution prepared in water was added to each well. Following preparation, the plates were incubated at the either 30 °C or 37 °C, as indicated, for 72 hours.

All dose-response assays were performed in biological triplicate with a minimum of three technical replicates. Growth, as determined by a reading of absorbance at 600 nm, was normalized to untreated controls and was corrected for the absorbance of 4456dh in the YPD medium. The minimum inhibitory concentration causing 80% growth reduction, or MIC_{80}, was determined based on the OD_{600} values (5, 6). Growth was plotted as a heat map using Excel.

Fungicidal Assays

Cells previously exposed to compounds for 48 or 72 hours in dose-response matrices were spotted to test the viability of treated cells. 3 µL was extracted from each well with a multi-channel pipette and dispensed onto drug-free YPD agar plates. Plates were incubated at 30 °C for 48 hours and photographed. All experiments were performed in biological and technical duplicate.

Trehalose Measurement

Candida and *Cryptococcus* strains were grown in liquid cultures at 30 °C and then 42 °C or 37 °C, respectively. Cells at each temperature were exposed to either water (vehicle control) or 0.5 mM 4456dh. The OD_{600} was determined followed by dilution of overnight cultures to an equivalent of 0.1 OD_{600} of cells. Cells were pelleted, frozen and lyophilized and stored in -80 °C. Pellets were lysed by vortexing with sterile glass beads. The cell-free extract was generated by adding 1X PBS and centrifuging the tubes to pellet the glass beads. The supernatant containing cell-free extracts was exposed to trehalase (Sigma) overnight at 37 °C and then tested for trehalose levels according to the Glucose Assay Kit (MAK476, Sigma) protocol.

Zone of Inhibition Assays

Strains were grown in overnight cultures in YPD (yeast-peptone-dextrose) medium at 30 °C. Optical density (OD_{600}) was determined with an Infinite PRO plate reader (Tecan). OD_{600} was adjusted to 0.01 (10^6 cells/mL) through dilution with YPD, and 200 µL diluted culture from each strain was plated onto YPD plates and spread using sterile beads (Sigma). After the plates had dried, a single disk soaked with either 15 µL of water, 4456dh or 1 µg/mL FK506 disk (6 mm diameter, Becton, Dickinson and Company) was placed in a quadrant of each plate. FK506 was used as the positive control (7). Plates were incubated at either 30 °C or 37 °C for 48 h and then imaged.

***C. albicans* Filamentation Assays**

C. albicans wild-type strain SC5314 and respective *tps1*/*tps1* deletion strains were grown overnight at 30°C in 5 ml liquid YPD. 1 OD equivalent of the overnight grown cells were harvested, resuspended in 1 ml of YPD+10% fetal bovine serum and incubated at 37°C for 2 hours. The wild-type culture was grown in four replicates and three of them were treated with Lee4456dh at

different concentrations (0.5mM, 1mM, and 5mM) and one was used as untreated control. *tps1*/*tps1* deletion mutant was used as a negative control for filamentation in these assays. 10 µl of cell suspension was directly used for imaging the filamentation status of the cells using the Zeiss Axio Scope microscope attached to the Axiocam at 40X magnification. The images were processed using Fiji (8) for the final presentation.

Epsilometer Test (E-test)

C. neoformans wild type (H99) was incubated in a shaking culture of YPD medium for 18 h at 30ºC. The culture was diluted to an OD_{600} 0.6, diluted 10-fold in PBS, and 100 µL was spread onto 60 X 15 mm YPD agar plates prepared with different concentrations of 4456dh. After the surface was completely dry, an E-test strip containing a gradient of fluconazole (Biomérieux, Marcy-l'Étoile, France) was applied to each plate. The plates were incubated at 37 °C and imaged after 72 h.

C. neoformans Capsule Assays

Capsule was induced by inoculating fresh *C. neoformans* wild-type strain H99 cells in CO_2-independent liquid media (Gibco) for 72 hours at 30 °C. The wild-type culture was grown in four replicates and three of them were treated with Lee4456dh at different concentrations (0.1 mM, 0.5 mM and 1 mM) and one was used as untreated control. The *C. neoformans tps1*Δ mutant was used as a negative control for capsule formation. For visualization, BactiDrop India Ink (Remel) was added to the cell suspension and 5 µL of this mixture was then spotted onto a glass slide. Capsule was observed with a Zeiss Axio Imager A1 fluorescence microscope with a 100X objective. Images were taken with an AxioCam MRm digital camera with ZEN Pro software (Zeiss). The size of the capsule of > 100 cells per 4456dh treatment was quantified using Image J software (https://imagej.net/ij/index.html) by measuring the width of the halo created by capsule and made visible with the negative India Ink staining.

Supplemental Figure Legends

Supplemental Figure 1. Fluorescence polarization dose-response curves. Fluorescence polarization binding curves of hits from the 3-point pharmacophore (3PP) library. Two wells per data point. Error bars represent standard error.

Supplemental Figure 2. A structure-guided approach led to the derivatization of SJ6675. **A)** CaTps1-SJ6675 crystal structure at resolution of 3.5 Å. The zoomed-in view shows SJ6675 in the proximity of CaTps1 substrate-binding residues R280, K285, N382 and L383. **B)** The chemical structures and identities of the SJ6675 derivative library. 4456, the focus of additional studies presented here, is highlighted. **C)** Effect of derivatives on the activity of 6xHis-CaTps1. CaTps1 enzymatic activity was tested using a coupled activity assay and normalized to the DMSO control (data represent the mean ± SEM, n = 3). Compounds that precipitated are represented with the grey bars. The compounds that did not precipitate and the DMSO vehicle control are represented as purple and blue, respectively.

Supplemental Figure 3. 4456dh NMR validation and elemental analysis. A) ^{1}H NMR spectra were recorded on a Bruker 400 MHz spectrometer. Chemical shifts (δ) are reported in parts per million relative to the residual solvent peak or internal standard. Coupling constants (J) are reported in hertz (Hz). **B)** Elemental analysis confirmed the chemical composition of C, H, N and Cl elements in 4456dh.

Supplemental Figure 4. Polder omit map of 4456 in the CaTps1-4456 structure. A polder omit map, contoured at 3σ (purple mesh), shows 4456 electron density bound in the CaTps1 substrate-binding site. CaTps1 residues and 4456 are shown as atom-colored stick and ball representations.

Supplemental Figure 5. Residues within 4 Å of 4456 in the *C. albicans* Tps1-4456 complex are conserved. Tps1 sequences of *C. gattii*, *C. deneoformans*, *C. neoformans*, *A. fumigatus*, *C. glabrata*, *C. auris* and *C. albicans* are aligned. The sequences are taken from the NCBI database. Key residues within 4 Å of 4456 are highlighted by red boxes. The numbering in the red text is based on the *C. albicans* Tps1 protein sequence.

Supplemental Figure 6. 4456 binds in the structurally conserved pocket of Tps1 enzymes. A) Structural alignments of one subunit of the Tps1 enzymes from *C. albicans* (pink, PDB: 5HUU), *E. coli* (purple, 1UQU), *C. neoformans* (green, 8FHW) and *A. fumigatus* (blue, 5HVM) with 4456 (sand) from the *C. albicans* Tps1-4456 complex modelled into the conserved catalytic pocket of Tps1 (1, 2, 9). **B)** A ConSurf analysis for CaTps1 and 4456 (as a grey, surface-representation). The amino acids are colored by their levels of conservation as shown with the color-coding bar. Teal represents amino acids that are variable, whereas dark magenta amino acids are highly conserved. Regions for which there are not enough data to form a conclusion are shown in yellow.

Supplemental Figure 7. 4456dh bioactivity at permissive growth temperatures (30 °C) for *Candida* and *Cryptococcus*. A) Table showing minimum inhibitory concentration values for 4456dh in *Candida* and *Cryptococcus* species grown at 30 °C. **B)** *Candida* spot assays show ability of cells to grow after exposure to 4456 and indicate fungicidal activity of 4456dh at 30 °C. **C)** *Cryptococcus* spot assays show ability of cells to grow after exposure to 4456 and indicate fungicidal activity of 4456dh at 30 °C.

Supplemental Figure 8. 4456dh retains activity in a *C. deneoformans tps1*Δ. A) Zone of inhibition assays with *C. deneoformans* strains JEC21 and JEC20 grown at 37 °C. **B)** Zone of inhibition assays with *C. deneoformans* strains JEC21 *tps1*Δ and JEC20 *tps1*Δ grown at 30 °C.

The circular graphic illustrates the compounds added to the sterile disc. FK506 (1 μg/mL), a calcineurin inhibitor, is the positive control and water is the vehicle control.

Supplemental Figure 9. Synergy between 4456dh and fluconazole in *C. neoformans*. Images of YPD plates containing serially-diluted concentrations of 4456dh with fluconazole E-test strips. The plate lacking 4456dh is the negative control. Zones of *C. neoformans* growth inhibition indicate the relative susceptibility of the fungi to the combination of drugs.

Supplemental Figure 10. 4456dh dose-dependent decrease in trehalose in *C. neoformans*. Trehalose accumulation measurements for *C. neoformans* cultured at 37 °C in the presence of 0.1, 0.5, 1.0 and 2.0 mM 4456dh. The untreated sample served as the negative control. Trehalose was measured using a colorimetric glucose assay after treatment of cultures with trehalase. Error bars represent standard error.

Supplemental Figure 11. Quantification of capsule formation in *C. neoformans* treated with 4456dh. Images taken were analyzed with ImageJ. A minimum of 100 cells per treatment or strain were analyzed. The median for each sample is indicated with the black line. Statistical analysis was performed in GraphPad Prism using the one-way ANOVA with Bartlett's posthoc test to compare the untreated sample to the individual treated samples. P values < 0.05 are indicated as significant on the graph.

Supplemental Figure 12. Selected predicted structures with structural homology to *C. albicans* Tps1. The query *C. albicans* Tps1 (PDB: 5HUU, Chain A) is shown in grey (1). AlphaFold predicted structures, identified by a hierarchical search of the DALI protein structure comparison server, are shown in ribbon representations and colored according to the pLDDT (predicted local distance difference test) (10).

Supplemental Tables

Supplemental Table 1. Data collection and refinement statistics of the structure of 6xHis-CaTps1 in complex with compound SJ6675.

**6xHis-CaTps1 - SJ6675 complex**	
Resolution range	47.05 - 3.501 (3.63 - 3.5)
Space group	$P\ 6_5\ 2\ 2$
Unit cell	115.1 115.1 266.8 90 90 120
Total reflections	157,931 (14,753)
Unique reflections	13,858 (1,326)
Multiplicity	11.4 (11.1)
Completeness (%)	99.60 (99.10)
Mean I/sigma(I)	17.15 (6.63)
Wilson B-factor	94.6
R-merge	0.0969 (0.2914)
R-meas	0.1017 (0.306)
R-pim	0.03055 (0.09207)
$CC_{1/2}$	0.999 (0.971)
CC*	1 (0.993)
Reflections used in refinement	13,814 (1324)
Reflections used for R-free	1,366 (133)
R-work	0.2300 (0.3096)
R-free	0.2748 (0.3868)
Number of non-hydrogen atoms	7,474
macromolecules	7,474
ligands	0
solvent	0
Protein residues	934
RMS(bonds)	0.003
RMS(angles)	0.64
Ramachandran favored (%)	96.34
Ramachandran allowed (%)	3.23
Ramachandran outliers (%)	0.43
Rotamer outliers (%)	0.00
Clashscore	6.65
Average B-factor	92.46
macromolecules	92.46

Statistics for the highest-resolution shell are shown in parentheses.

Supplemental Table 2. Similarity between *C. albicans* Tps1 and Alphafold predicted glycosyltransferase structures in *C. albicans*.

AF#	Z-score	RMSD	%ID	Protein
Q92410	47.8	1.1	64	Alpha, Alpha-trehalose phosphate synthase
A0A1D8PIS4	38.5	1.4	32	Trehalose 6-phosphate synthase/phosphatase
Q5AI14	37.0	1.7	35	Trehalose-phosphatase
Q59LF2	25.0	2.9	13	Alpha-1,3/1,6-mannosyltransferase Alg2
Q59Q79	21.8	3.6	11	Chitobiosyldiphosphodolichol beta mannosyltransferase
Q5A6R7	20.6	3.2	13	Glcnac-Pi Synthesis protein
Q59S72	16.3	3.2	9	GDP-Man:Man(3)GlcNAc(2)-PP-Dol alpha-1,2-mannosyltransferase Alg11
Q5A850	16.0	4.0	11	Glycogen synthase
A0A1D8PQQ3	13.8	3.7	9	Alpha-1,4 glucan phosphorylase
Q5A950	9.6	4.4	7	Sterol 3-beta-glucosyltransferase Atg26
Q5A868	8.5	4.6	9	Phosphopantothenoylcysteine decarboxylase complex subunit
Q5ABE5	8.3	4.6	10	UDP-N-acetylglucosamine transferase subunit ALG13
A0A1D8PQJ9	7.8	3.1	10	Ubiquinone biosynthesis protein
Q5A1C0	7.7	3.1	9	Uncharacterized protein
Q5AK46	7.5	4.2	6	Multifunctional fusion protein
Q5AML3	7.5	3.2	10	Oxidoreductase
A0A1D8PE77	7.4	4.8	7	Ubiquitin-activating enzyme E1-like
A0A1D8PKD2	7.3	5.2	7	Phosphopantothenoylcysteine decarboxylase complex subunit
A0A1D8PKJ3	7.3	10.3	3	E1 ubiquitin-activating protein UBA1
Q59XU5	7.2	3.4	6	Ras-like protein 1
A0A1D8PCN0	7.0	3.4	8	Bifunctional UDP-glucose 4-epimerase/aldose 1-epimerase
Q5AP65	7.0	3.4	11	Protein FMP52, mitochondrial
Q5A5N6	7.0	10.0	12	UDP-N-acetylglucosamine transferase subunit ALG14
A0A1D8PKJ4	6.9	3.1	13	Saccharopine dehydrogenase (NADP+, L-glutamate-forming)
A0A1D8PRL3	6.9	3.5	10	NAD(P)-bd domain-containing protein

A hierarchical search for query structure against the AlphaFold database using the DALI protein structure comparison server. The query *C. albicans* Tps1 (PDB:5HUU, Chain A) was used to search for similar predicted structures generated by AlphaFold in *C. albicans* (1).

Supplemental Table 3. Structures in the PDB that have similarity to *C. neoformans* Tps1 (PDB: 8FO1).

PDB	RMSD (Å)	%ID	Aligned Residues	Protein	Organism
6PTA	2.43	5	94	ARF family small GTPase ARF1 in complex with GDP	*C. albicans*
7RLL	2.46	10	93	ARF3 in complex with guanosine-3'-monophosphate-5'-diphosphate	*C. albicans*
8PHX	2.67	9	79	Receiver Domain of the Hybrid Histidine Kinase Sln1	*C. albicans*
5UF8	2.71	7	95	ARF family small GTPase ARF2 in complex with GDP	*C. albicans*
6EOA	3.23	10	125	Crystal Structure of HAL3	*C. neoformans*
6EFW	3.28	6	119	YjeF family protein	*C. neoformans*
6EFX	3.58	10	130	YjeF family protein in complex with AMPPNP	*C. neoformans*
8JAT	2.52	11	95	3-ketodihydrosphingosine reductase TSC10	*C. neoformans*

A search for query structure against the NCBI VAST+ Similar Structures search algorithm. The query *C. neoformans* Tps1 (PDB: 8FO1) was used to search for similar predicted structures found in the PDB (2).

Supplemental Table 4. Structures in the PDB that have similarity to *C. albicans* Tps1 (PDB: 5HUU).

PDB	RMSD (Å)	%ID	Aligned Residues	Protein	Organism
7RLL	2.52	7	97	ARF3 in complex with guanosine-3'-monophosphate-5'-diphosphate	*C. albicans*
7DLL	2.41	7	87	Short chain dehydrogenase 2 (SCR2) with NADPH	*C. parapsilosis*
7DMG	3.00	6	97	Short chain dehydrogenase 2 (SCR2) with NADP	*C. parapsilosis*
7VYQ	3.11	5	104	Short chain dehydrogenase (SCR) with NADP and ethyl 4-chloroacetoacetate	*C. parapsilosis*
7DLD	3.13	12	109	(S)-carbonyl reductases in different oligomerization states	*C. parapsilosis*
7DLM	3.20	7	104	Short chain dehydrogenase (SCR) with NADPH	*C. parapsilosis*
8JAT	2.65	12	98	3-ketodihydrosphingosine reductase TSC10	*C. deneoformans*

A search for query structure against the NCBI VAST+ Similar Structures search algorithm. The query *C. albicans* Tps1 (PDB: 5HUU) was used to search for similar predicted structures found in the PDB (1).

Supplemental Table 5. Comparison of Minimum Inhibitory Concentrations of 4456dh and clinically relevant antifungal therapeutics.

Drug	Pathogen	MIC_{80} (µg/mL)	Reference
4456dh	*C. albicans*	5160	This publication
4456dh	*C. auris*	2780	This publication
4456dh	*C. glabrata*	3.1	This publication
4456dh	*C. neoformans*	2780	This publication
4456dh	*C. deneoformans*	1190	This publication
4456dh	*C. gattii*	2780	This publication
Fluconazole	C. albicans	1.84	(11)
Voriconazole	C. albicans	0.06	(11)
Caspofungin	C. albicans	1	(12)
Amphotericin B	C. neoformans	2	(13)
Flucytosine	C. neoformans	8	(13)

Supplemental Table 6. Fungal strains used in this study.

Strain Name	Genotype	Source
C. albicans SC5314	Wild-type	(14)
C. auris B11220	AR0381	(15)
C. glabrata CBS138	Wild-type	(16)
C. neoformans H99α	Wild-type *MAT*α	(17)
C. deneoformans JEC20	Wild-type *MATa*	(18)
C. deneoformans JEC21	Wild-type *MAT*α	(18)
C. gattii R265	*C. gattii* wild-type VGII mating type α	(19)
C. deneoformans JEC20 *tps1*Δ	MATa*tps1*::*NAT*	(20)
C. deneoformans JEC21 *tps1*Δ	MATα *tps1*::*NAT*	(20)

Supplemental References

1. Miao Y, Tenor JL, Toffaletti DL, Maskarinec SA, Liu J, Lee RE, Perfect JR, Brennan RG. 2017. Structural and In Vivo Studies on Trehalose-6-Phosphate Synthase from Pathogenic Fungi Provide Insights into Its Catalytic Mechanism, Biological Necessity, and Potential for Novel Antifungal Drug Design. mBio 8.

2. Washington EJ, Zhou Y, Hsu AL, Petrovich M, Tenor JL, Toffaletti DL, Guan Z, Perfect JR, Borgnia MJ, Bartesaghi A, Brennan RG. 2024. Structures of trehalose-6-phosphate synthase, Tps1, from the fungal pathogen *Cryptococcus neoformans*: A target for antifungals. Proc Natl Acad Sci U S A 121:e2314087121.

3. Eschenfeldt WH, Lucy S, Millard CS, Joachimiak A, Mark ID. 2009. A family of LIC vectors for high-throughput cloning and purification of proteins. Methods Mol Biol 498:105–115.

4. Errey J, Lee S, Gibson R, Fleites C, Barry C, Jung P, O'Sullivan A, Davis B, Davies G. 2010. Mechanistic insight into enzymatic glycosyl transfer with retention of configuration through analysis of glycomimetic inhibitors. Angew Chem Int Ed Engl 49:1234–7.

5. Puumala E, Zaslaver O, Chen A, Duncan D, Fogal M, Shapiro RS, Mazhab-Jafari MT, Whitesell L, Montenegro-Burke JR, Robbins N, Cowen LE. 2022. The Trisubstituted Isoxazole MMV688766 Exerts Broad-Spectrum Activity against Drug-Resistant Fungal Pathogens through Inhibition of Lipid Homeostasis. mBio 13:e0273022.

6. Xie L, Singh-Babak SD, Cowen LE. 2012. Minimum Inhibitory Concentration (MIC) Assay for Antifungal Drugs. Bio-protocol 2:e252.

7. Lee Y, Lee K-T, Lee SJ, Beom JY, Hwangbo A, Jung JA, Song MC, Yoo YJ, Kang SH, Averette AF, Heitman J, Yoon YJ, Cheong E, Bahn Y-S. 2018. *In Vitro* and *In Vivo*

Assessment of FK506 Analogs as Novel Antifungal Drug Candidates. Antimicrob Agents Chemother 62.

8. Schindelin J, Arganda-Carreras I, Frise E, Kaynig V, Longair M, Pietzsch T, Preibisch S, Rueden C, Saalfeld S, Schmid B, Tinevez J-Y, White DJ, Hartenstein V, Eliceiri K, Tomancak P, Cardona A. 2012. Fiji: an open-source platform for biological-image analysis. Nature Methods 9:676–682.

9. Gibson RP, Tarling CA, Roberts S, Withers SG, Davies GJ. 2004. The donor subsite of trehalose-6-phosphate synthase: binary complexes with UDP-glucose and UDP-2-deoxy-2-fluoro-glucose at 2 A resolution. J Biol Chem 279:1950–1955.

10. Jumper J, Evans R, Pritzel A, Green T, Figurnov M, Ronneberger O, Tunyasuvunakool K, Bates R, Žídek A, Potapenko A, Bridgland A, Meyer C, Kohl SAA, Ballard AJ, Cowie A, Romera-Paredes B, Nikolov S, Jain R, Adler J, Back T, Petersen S, Reiman D, Clancy E, Zielinski M, Steinegger M, Pacholska M, Berghammer T, Bodenstein S, Silver D, Vinyals O, Senior AW, Kavukcuoglu K, Kohli P, Hassabis D. 2021. Highly accurate protein structure prediction with AlphaFold. Nature 596:583–589.

11. Azevedo AC, Bizerra FC, da Matta DA, de Almeida LP, Rosas R, Colombo AL. 2011. *In vitro* susceptibility of a large collection of *Candida* Strains against fluconazole and voriconazole by using the CLSI disk diffusion assay. Mycopathologia 171:411–416.

12. Kartsonis N, Killar J, Mixson L, Hoe C-M, Sable C, Bartizal K, Motyl M. 2005. Caspofungin susceptibility testing of isolates from patients with esophageal candidiasis or invasive candidiasis: relationship of MIC to treatment outcome. Antimicrob Agents Chemother 49:3616–3623.

13. Archibald LK, Tuohy MJ, Wilson DA, Nwanyanwu O, Kazembe PN, Tansuphasawadikul S, Eampokalap B, Chaovavanich A, Reller LB, Jarvis WR, Hall GS, Procop GW. 2004. Antifungal susceptibilities of *Cryptococcus neoformans*. Emerg Infect Dis 10:143–145.

14. Jones T, Federspiel NA, Chibana H, Dungan J, Kalman S, Magee BB, Newport G, Thorstenson YR, Agabian N, Magee PT, Davis RW, Scherer S. 2004. The diploid genome sequence of *Candida albicans*. Proc Natl Acad Sci U S A 101:7329–7334.

15. Lutgring JD, Machado M-J, Benahmed FH, Conville P, Shawar RM, Patel J, Brown AC. 2018. FDA-CDC Antimicrobial Resistance Isolate Bank: a Publicly Available Resource To Support Research, Development, and Regulatory Requirements. J Clin Microbiol 56.

16. Dujon B, Sherman D, Fischer G, Durrens P, Casaregola S, Lafontaine I, de Montigny J, Marck C, Neuvéglise C, Talla E, Goffard N, Frangeul L, Aigle M, Anthouard V, Babour A, Barbe V, Barnay S, Blanchin S, Beckerich J-M, Beyne E, Bleykasten C, Boisramé A, Boyer J, Cattolico L, Confanioleri F, de Daruvar A, Despons L, Fabre E, Fairhead C, Ferry-Dumazet H, Groppi A, Hantraye F, Hennequin C, Jauniaux N, Joyet P, Kachouri R, Kerrest A, Koszul R, Lemaire M, Lesur I, Ma L, Muller H, Nicaud J-M, Nikolski M, Oztas S, Ozier-Kalogeropoulos O, Pellenz S, Potier S, Richard G-F, Straub M-L, Suleau A, Swennen D, Tekaia F, Wésolowski-Louvel M, Westhof E, Wirth B, Zeniou-Meyer M, Zivanovic I, Bolotin-Fukuhara M, Thierry A, Bouchier C, Caudron B, Scarpelli C, Gaillardin C, Weissenbach J, Wincker P, Souciet J-L. 2004. Genome evolution in yeasts. Nature 430:35–44.

17. Perfect JR, Ketabchi N, Cox GM, Ingram CW, Beiser CL. 1993. Karyotyping of *Cryptococcus neoformans* as an epidemiological tool. J Clin Microbiol 31:3305–3309.

18. Kwon-Chung KJ, Edman JC, Wickes BL. 1992. Genetic association of mating types and virulence in *Cryptococcus neoformans*. Infect Immun 60:602–605.

19. Kidd SE, Hagen F, Tscharke RL, Huynh M, Bartlett KH, Fyfe M, Macdougall L, Boekhout T, Kwon-Chung KJ, Meyer W. 2004. A rare genotype of *Cryptococcus gattii* caused the cryptococcosis outbreak on Vancouver Island (British Columbia, Canada). Proc Natl Acad Sci U S A 101:17258–17263.

20. Lin X, Heitman J. 2005. Chlamydospore formation during hyphal growth in *Cryptococcus neoformans*. Eukaryot Cell 4:1746–1754.
